# Integrating community-based HIV and non-communicable disease care with microfinance groups: a feasibility study in Western Kenya

**DOI:** 10.1186/s40814-022-01218-6

**Published:** 2022-12-28

**Authors:** Catherine Kafu, Juddy Wachira, Victor Omodi, Jamil Said, Sonak D. Pastakia, Dan N. Tran, Jael Adongo Onyango, Dan Aburi, Marta Wilson-Barthes, Omar Galárraga, Becky Lynn Genberg

**Affiliations:** 1 Academic Model Providing Access to Healthcare, P.O. Box 4606-30100, Eldoret, Kenya; 2 School of Literature, Language and Media, Department of Media Studies, University of Witwatersrand, 1 Jan Smuts Avenue, Braamfontein, Johannesburg, 2000 South Africa; 3 School of Medicine, Department of Behavioral Science, Moi University College of Health Sciences, P.O. Box 4606-30100, Eldoret, Kenya; 4 School of Medicine, Department of Human Anatomy, Moi University College of Health Sciences, P.O. Box 4606-30100, Eldoret, Kenya; 5 Center for Health Equity and Innovation, Purdue University College of Pharmacy, 640 Eskenazi Ave, Indianapolis, IN 46202 USA; 6 Department of Pharmacy Practice, Temple University School of Pharmacy, 3307 N Broad St, Philadelphia, PA 19140 USA; 7 Department of Epidemiology, Brown University School of Public Health, 121 South Main Street, Providence, RI 02912 USA; 8 Department of Health Services, Policy and Practice, Brown University School of Public Health, 121 South Main Street, Providence, RI 02912 USA; 9 Department of Epidemiology, Johns Hopkins Bloomberg School of Public Health, 615 N. Wolfe Street, Baltimore, MD 21205 USA

**Keywords:** Differentiated care, Human immunodeficiency viruses (HIV), Non-communicable diseases, Community-based care, Microfinance, Feasibility study, Randomized controlled trial, Implementation science

## Abstract

**Background:**

The Harambee study is a cluster randomized trial in Western Kenya that tests the effect, mechanisms, and cost-effectiveness of integrating community-based HIV and non-communicable disease care within microfinance groups on chronic disease treatment outcomes. This paper documents the stages of our feasibility study conducted in preparation for the Harambee trial, which include (1) characterizing the target population and gauging recruitment capacity, (2) determining community acceptability of the integrated intervention and study procedures, and (3) identifying key implementation considerations prior to study start.

**Methods:**

Feasibility research took place between November 2019 and February 2020 in Western Kenya. Mixed methods data collection included surveys administered to 115 leaders of 105 community-based microfinance groups, 7 in-person meetings and two workshops with stakeholders from multiple sectors of the health system, and ascertainment of field notes and geographic coordinates for group meeting locations and HIV healthcare facilities. Quantitative survey data were analyzed using STATA IC/13. Longitude and latitude coordinates were mapped to county boundaries using Esri ArcMap. Qualitative data obtained from stakeholder meetings and field notes were analyzed thematically.

**Results:**

Of the 105 surveyed microfinance groups, 77 met eligibility criteria. Eligible groups had been in existence from 6 months to 18 years and had an average of 22 members. The majority (64%) of groups had at least one member who owned a smartphone. The definition of “active” membership and model of saving and lending differed across groups. Stakeholders perceived the community-based intervention and trial procedures to be acceptable given the minimal risks to participants and the potential to improve HIV treatment outcomes while facilitating care integration. Potential challenges identified by stakeholders included possible conflicts between the trial and existing community-based interventions, fear of group disintegration prior to trial end, clinicians’ inability to draw blood for viral load testing in the community, and deviations from standard care protocols.

**Conclusions:**

This study revealed that it was feasible to recruit the number of microfinance groups necessary to ensure that our clinical trial was sufficient powered. Elicitation of stakeholder feedback confirmed that the planned intervention was largely acceptable and was critical to identifying challenges prior to implementation.

**Trial registration:**

The original trial was prospectively registered with ClinicalTrials.gov (NCT04417127) on 4 June 2020.

**Supplementary Information:**

The online version contains supplementary material available at 10.1186/s40814-022-01218-6.

## Key messages regarding feasibility study

- This article describes a feasibility assessment done in advance of a cluster randomized trial among patients living with HIV and non-communicable disease in Western Kenya [ClinicalTrials.gov Identifier: NCT04417127]. To determine trial feasibility, we assessed (1) whether there were a sufficiently high number of established, eligible microfinance groups interested in trial enrollment as well as the geographic accessibility of the groups, and (2) whether group dynamics would support long-term trial engagement. We additionally worked to educate and garner support from local health ministry personnel and other multi-sectoral stakeholders surrounding trial objectives.
- Of the 105 community-based microfinance groups that were surveys, 77 met the trial’s eligibility criteria. Among eligible groups, we found differences in terms of the definition of active membership, microfinance (e.g., saving and lending) models, and meeting schedule. If shown to be effective, stakeholders expressed a desire to have the trial’s community-based care intervention integrated within their county’s existing care programs to promote continuity of care for trial participants.
- The feasibility work confirmed some of the study’s original implementation strategies such as the ability to implement established facility-based HIV and non-communicable disease care protocols in the community. However, it also facilitated important changes to the study design including revisions to the trial’s inclusion criteria at both the group- and individual-level, inclusion of additional care providers in intervention delivery, and the importance of researchers collaborating with established care programs to coordinate patient care during each stage of the trial.

## Background

Despite gains in antiretroviral therapy (ART) coverage for people living with HIV (PLHIV) and reductions in HIV-related morbidity and mortality, significant gaps remain. Retention in care remains a challenge in sub-Saharan Africa [1] where only 50% of PLHIV are virally suppressed [2]. For individuals living with HIV, transportation barriers, food insecurity, and lack of social support are some of the factors that contribute to poor viral suppression and retention in care [3]. At the facility level, disproportionate provider-patient ratio, poor provider-patient dynamics, and inefficient vertical care delivery further contribute to poor health outcomes among PLHIV [4, 5]. Strengthening effective, multi-level interventions that can address these social and economic barriers to viral suppression is critical to achieving the 90-90-90 WHO goals [6].

As PLHIV are living longer due to ART, the burden of non-communicable diseases (NCDs) among PLHIV is also increasing [7–10]. This necessitates the integration of NCD care into established HIV treatment platforms in order to collectively address the increasing number of HIV patients requiring NCD care [9, 11, 12]. In Kenya, integrated community-based HIV, diabetes and hypertension programs have previously been effectively delivered within medication adherence clubs [13, 14], though the impact of this differentiated care approach on clinical outcomes has not been well documented [13, 14]. Thus, robust evidence of the effect of integrated HIV/NCD care on patient health outcomes is needed to inform policy planning and scale up.

For individuals who experience poverty-related barriers to care, offering differentiated care in tandem with economic strengthening may improve the effectiveness and longer-term sustainability of integrated care models. The literature suggests that people with chronic conditions including HIV, hypertension, and diabetes who participate in microfinance and other income-generating activities have better retention in care and improved treatment outcomes compared to individuals who do not engage in economic strengthening [15–17]. Participation in group-based rather than individual microfinance can serve as a mechanism for both economic and social support, which is especially important for PLHIV who experience disease-related stigma and psychological barriers that threaten treatment adherence [16, 18, 19]. The limited data show that providing medical visits for HIV and non-communicable diseases within existing microfinance groups is associated with better treatment outcomes [17, 20]. However, because microfinance groups can suffer from group dissolution and ethical concerns related to group exclusions, evidence of effective implementation science approaches for sustaining group-based care models is also needed [21, 22].

To address this critical evidence gap, we are conducting a randomized control trial—*Harambee: Integrated Community-based HIV/NCD Care and Microfinance Groups in Kenya* (ClinicalTrials.gov Identifier: NCT04417127)—to demonstrate the effectiveness and sustainability of an innovative differentiated HIV and NCD care delivery model. In light of the rapidly growing burden of non-communicable disease among PLWHIV in sub-Saharan Africa [23–26], the *Harambee* trial aims to collectively improve patient HIV treatment outcomes while addressing non-communicable chronic disease care needs within community-based microfinance groups [27]. The specific aims of the *Harambee* study are to (1) evaluate the extent to which integrated community-based HIV care with group microfinance affects retention in care and viral suppression among PLHIV in rural Western Kenya, (2) identify specific mechanisms through which microfinance and integrated community-based care impact viral suppression, and (3) assess the cost-effectiveness of microfinance and integrated community-based care delivery to maximize future policy and practice relevance of this promising intervention strategy. By providing rigorous evidence of the effectiveness and cost-effectiveness of a novel integrated community-based care model, the trial will respond to calls for integrated responses that address the economic and treatment needs of persons living with HIV and NCDs in LMICs [26–29].

This paper describes the methods used to conduct a feasibility study in preparation for the start of the *Harambee* cluster randomized trial. Feasibility research is an essential component of rigorous implementation science with researchers arguing for its value prior to conducting randomized controlled trials (RCTs) [30]. Conducting a feasibility study prior to a large RCT is likely to enhance effectiveness of research [31]. Drawing on the main objectives of feasibility studies proposed by Orsmond and Cohn [31], the goals of this research were to determine (1) the recruitment capability and sample characteristics, (2) the acceptability and suitability of the intervention and study procedures, and (3) key implementation considerations for the study and intervention.

## Methods

### Setting

The Academic Model Providing Access To Healthcare (AMPATH) is a joint partnership between Moi University School of Medicine, Moi Teaching and Referral Hospital, and a consortium of universities and academic medical centers in North America [32]. Established as an HIV care program, AMPATH currently serves over 150,000 HIV-positive patients in 800 government-supported health facilities across 10 counties in Western Kenya. All HIV care and treatment is provided free of charge [33]. AMPATH, in cooperation with the Kenyan Health Ministry, emphasizes a comprehensive, integrated, community-centered, and financially sustainable health care delivery approach that is responsive to the needs of the entire population [32]. First, in response to the substantial and growing burden of diabetes mellitus and hypertension, AMPATH formed a chronic disease management (CDM) program [34, 35] and a reliable supply chain system for hypertension and diabetes medications [36]. Second, to address patient’s economic security needs, AMPATH created the Family Preservation Initiative (FPI) which supports patients to earn a sustainable source of income through skills training, microcredit, agribusiness support, a fair-trade-certified crafts workshop and agricultural cooperatives [37]. FPI has formed more than 1349 community-based microfinance groups (with over 27,249 group members) as part of *group integrated savings for empowerment* (GISE) program. FPI personnel work with trained group empowerment service providers (GESPs) at the community level to form, train and continually offer needed support to the community-based GISE groups. GISE group members mobilize and manage savings and provide interest-bearing loans to members without a requirement for collateral. All members of the group are encouraged to save some money at each meeting and contribute a nominal fee to a social fund that supports unanticipated emergencies or welfare issues of group members [17]. Initially including only patients living with HIV, GISE groups have expanded to include pregnant women [38], patients with diabetes and hypertension [17, 34], and other community members living in AMPATH’s catchment areas. GISE groups offer a unique opportunity to provide critical HIV and NCD care to new, rural populations with minimal financial impact [39]. The established track record of effective collaboration between the aforementioned AMPATH programs offers an ideal setting for which to test our integrated community-based care model (described in subsequent sections) and to respond to the urgent needs of patients living with HIV and co-morbidities in Kenya’s most rural areas [40–43].

### Harambee study design

**Fig. 1:**
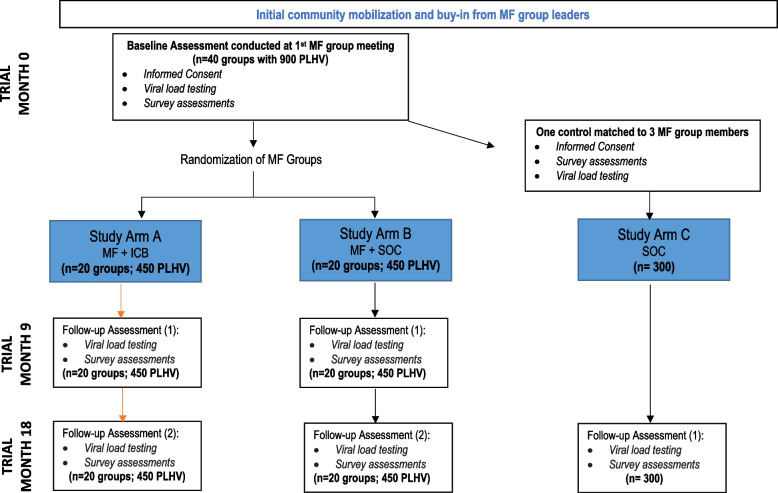
Schema for the Harambee cluster randomized trial

The Harambee study uses a cluster randomized design to perform a pragmatic evaluation of integrated community-based HIV and non-communicable disease care incorporated into existing microfinance groups located in Busia and Trans Nzoia counties of Western Kenya. Figure 1 presents the design of the three-arm cluster randomized trial.

Participants randomized to the intervention arm (*study arm A*) will receive the integrated community-based care (ICB) intervention during regularly scheduled microfinance group meetings. The intervention will include (1) integrated care visits by clinical teams which will entail vital signs screening, consultation with a clinical officer, medication distribution (ART for HIV and medications for other chronic and acute conditions), and point-of-care laboratory testing (creatinine, blood glucose, hemoglobin A1C, and viral load), (2) peer support during regularly scheduled microfinance meetings, and (3) referrals to facilities for emergency or acute care needs that cannot be addressed in the community. *Study arm B* participants will meet as usual in their MF groups and will continue to receive regular standard of care from an AMPATH-supported facility. *Study arm C* participants will continue to receive regular standard of care from an AMPATH facility and will have had no exposure to an AMPATH microfinance group since enrolling in care.

### Feasibility study design

For this study, we implemented a mixed-methods approach to understand assess the feasibility of testing the integrated care intervention in the community via the planned randomized trial [44]. In the quantitative phase of this research, a survey was conducted to determine the recruitment capability and sample characteristics [31]. During the qualitative phase, field-notes and stakeholder meetings were used to establish the acceptability and suitability of the intervention and study procedures as well as the implementation considerations for the study and intervention [31]. Data collection occurred between November 2019 and February 2020.

### Data collection

#### Quantitative surveys with microfinance group leaders

Based on information provided by team members from AMPATH’s Family Preservation Initiative, we purposively sampled 36 GISE groups (13 in Busia; 23 in Trans Nzoia) during the first phase of the group mapping exercise which took place in November 2019. Microfinance groups were eligible to complete the survey if they met the following criteria: (a) had over 70% of members who were living with HIV, (b) were actively engaging in microfinance activities, (c) had met at least once in the last 6 months, (d) were comprised of members who received HIV care at an AMPATH health facility, and (e) was not currently enrolled in the AMPATH Community ART Group program. The second phase of group mapping took place between January and February 2020. During this phase, information from the AMPATH retention department and Community Health Volunteers (CHVs) working in each county’s AMPATH catchment area was used to purposively sample an additional 69 (39 in Busia; 30 in Trans Nzoia) community-based HIV support groups that engage in microfinance activities.

Personnel from the FPI program and AMPATH’s retention department introduced GESPs and CHVs to research team members. GESPs and CHVs then facilitated the introduction of our research team to GISE and community-based HIV support groups, providing contact information for microfinance group leaders (i.e., the chairperson, secretary, and treasurer of consenting groups). We worked with GESPs and CHVs on basis of their knowledge, experience, and good working relationship with GISEs and community-based HIV support groups respectively. The GESPs and CHVs introduced the study’s research assistants to the group leaders and an interview date was set. Verbal consent was obtained and researcher-based questionnaires were administered to the group lead. The group chairperson was the preferred candidate to respond to the survey; the questionnaire was administered to the group secretary or treasurer in stances when the chairperson was unavailable. We selected microfinance group leads because they are the key liaison and main contact person for group members and have a detailed understanding of group activities. A semi-structured questionnaire (Appendix 1) developed by the research team was programmed into REDCap®. This 16-question survey focused on three main domains: group characteristics, microfinance activities, and HIV care experience of group members.

The surveys were conducted by trained research assistants at the group’s meeting location for the purposes of retrieving the latitude and longitude coordinates of group meeting locations. To ensure privacy and confidentiality, the surveys were conducted in the group’s meeting room with only the research assistant and group lead present. Upon completion of the interview, the research assistants traveled to the health facility where the majority of group members receive HIV care in order to collect the facility’s geographic coordinates. Only one facility per group was entered into REDCap®. Geographic coordinates were then used to examine the distance between the group meeting location and the health facilities where members seek HIV care services. Each survey was administered in Kiswahili and lasted approximately 30–45 min.

#### Field notes

Additional field notes were taken during the survey sessions to record any information that could not be captured via REDCap®. Field notes recorded items that could potentially affect study implementation such as topography, issues of transportation to meeting locations, and other observed challenges and opportunities.

#### Stakeholder meetings

We conducted a series of 28 in-person meetings with stakeholders from multiple sectors of the health system (Table 1). Criterion sampling [45] was used to identify persons who would ensure the successful implementation of the intervention. These key stakeholders included the AMPATH care program management, AMPATH Chronic Disease program management, AMPATH care program county-based teams; county-based Ministry of Health officers, and personnel from donor-funded HIV programs working in the target counties. Initial face-to-face meetings introduced stakeholders to the study aims and scope. Follow-up meetings focused on updating the stakeholders on the progress of the group mapping including the successes and challenges. The AMPATH care program team in each county was invited to a workshop with discussions centered on: feedback from the survey, review of the intervention components and design, and review of protocols and data collection tools for the randomized trial. All meeting proceedings were documented in writing.

**Table 1:** Feasibility study data collection methods

Feasibility objective	Data collection method	Respondent type	No. conducted (no. respondents)	Key indicators ascertained
Recruitment capability and sample characteristics	Eligibility Survey	MF group leads	105 (105)	Number of MF groups Number of eligible MF groups Number of eligible members in MF groups Frequency of meetings for eligible MF groups Meeting location for eligible MF groups
Acceptability and suitability of the intervention and study procedures	Stakeholders meetings	County stakeholders	7 (63)	Perception of the intervention design
	Stakeholder workshops	Health facility stakeholders	2 (29)	
	Eligibility survey	Microfinance group leads	115 (115)	
Implementation considerations for the study and intervention	Stakeholders meetings	County stakeholders	7 (63)	Recommendations for the intervention design
	Stakeholder workshops	Health facility stakeholders	2 (29)	
	Eligibility survey	Microfinance group leads	115 (115)	

### Analysis

Quantitative survey data were analyzed using STATA IC/13 (College Station, TX: Stata Press). Longitude and latitude coordinates of the microfinance groups were mapped to county boundaries using Esri ArcMap. Qualitative data obtained from the meetings and field notes were analyzed thematically, with the themes derived directly from the data. Quantitative and qualitative data were triangulated to make strategic decisions about the study design and materials.

## Results

### Characterizing community-based microfinance groups and determining group eligibility

We surveyed 105 community-based microfinance groups, 53 groups in Trans Nzoia County and 52 groups in Busia County. Eighty-five percent of groups were comprised of a majority (≥ 70%) of members who were living with HIV. Nearly all (98%) of the HIV-positive group members sought HIV care at AMPATH health facilities, with only 1% of surveyed groups being enrolled in the Community ART Group care model at AMPATH.

Most (96%) of the groups were actively engaging in microfinance activities and 87% of the groups had met at least once in the last 6 months at the time of the survey. Only 9 groups had previously participated in a research study. Table 2 summarizes the group characteristics related to the trial’s inclusion criteria. Overall, 77 groups were eligible for the study: 44 groups in Busia County and 33 in Trans Nzoia County.

**Table 2:** Eligibility characteristics of surveyed microfinance groups

	Trans Nzoia	Busia
Community-based groups	*n* = 53	*n* = 52
Engagement in microfinance activities, *n* (%)	49 (78)	52 (100)
≥ 70% of group members are living with HIV, *n* (%)	38 (60)	51 (98)
≥ 1 group member owns a smartphone, *n* (%)	30 (48)	35 (67)
Previously participated in research, *n* (%)	1 (2)	8 (15)
Currently enrolled in an AMPATH CAG, *n* (%)	0 (0)	1 (2)
Eligible groups, *n* (%)	33 (62)	44 (85)

*AMPATH* Academic Model Providing Access to Healthcare, *CAG* community ART group

During the first phase of the survey, we found low HIV status disclosure at the group level within GISEs. This was especially true for Trans Nzoia County where only 11 out of the 23 surveyed GISE groups had at least 70% of the members disclosing their HIV status to the group. Given that the GISE program had begun as an initiative to financially empower HIV-positive patients, we expected to find high levels of HIV disclosure within groups. However, the GESPs revealed that most of the original GISE groups either evolved into mixed groups (i.e., groups with both HIV-positive and HIV-negative individuals) or were no longer active. Within mixed groups, members living with HIV frequently confide in GESPs/CHVs who act as a link between members and the health facility but they do not necessarily disclose their HIV status to other group members. Non-disclosure at the group level was attributed to high levels of stigma and discrimination towards people living with HIV in the community. The majority of group members reportedly chose to keep their HIV status confidential to maintain group cohesion, as most group members do not want to be associated with HIV. Thus, HIV disclosure within mixed groups was identified as a key factor that could potentially influence group sustainability during the trial. Overall groups reported a well-defined leadership structure at the group level.

### Group dynamics

After identifying the 77 groups that were eligible for the trial, it was important to understand their group dynamics. We were interested in understanding how the groups define active membership, the type and frequency of microfinance activities they engage in, and distance of the group meeting location in relation to the health facility where majority of the group members usually seek HIV care.

#### Defining group membership

Microfinance groups had an average of 22 members per group with approximately 17 active members at any one time. Groups define active membership differently; however, these definitions can be broadly categorized in three ways. First, an active member could be defined as one who attends all scheduled group meetings, remits their savings, takes up loans and repays loans in a timely manner. Second, an active member can also refer to someone one who attends up to 50% of group meetings and remits their savings or loan repayment in a timely manner. Third, active membership can also include a member who remits their savings and loan repayment without necessarily attending any group meetings (Table 3). Other groups had a special category of group members who were not defined as active/inactive given that their the sole purpose in the group is to repay a loan that they had defaulted on. Understanding inter-group differences in defining active membership was used to refine the Harambee trial’s inclusion criteria for both group and individual participants. While the group inclusion criteria can be based on factors related to the activity of the group as an entity, individual members must also meet specific inclusion criteria in order to be enrolled in the study. If the inclusion criteria allow for variable definitions of individual participation in the group, there would be concerns regarding whether or not individual participants were being exposed to the same intervention across the arms of the study.

**Table 3:** Active membership definitions

Definition of active membership	Number of microfinance groups using this definition	
	Trans Nzoia County (***n*** = 33)	Busia County (***n*** = 44)
An active member:		
– Attends all the group meetings	21	32
– Remits their savings		
– Takes up loans		
– Repays the loan in a timely manner		
An active member:		
– Attends up to 50% of the group meetings	9	12
– Remits their savings		
– Takes up loans		
– Repays the loan in a timely manner		
An active member:		
– Remits their savings	3	0
– Takes up loans		
– Repays the loan in a timely manner		

### Group financial activities

**Fig. 2:**
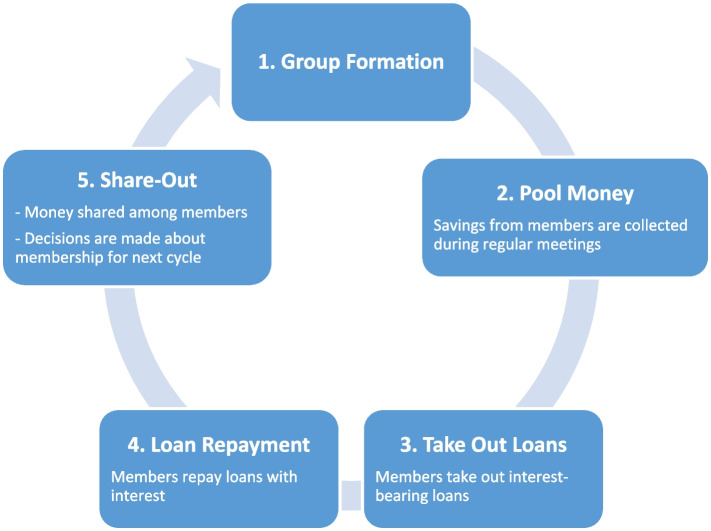
Table banking microfinance model

Eligible groups had been in existence for a period ranging from 6 months to 18 years at the time of the survey. All surveyed groups have an annual cycle period during which they engage in various activities, with microfinance being the dominant group activity. Groups follow diverse microfinance models. The most common microfinance model across the two counties is the table-banking system where group members contribute an agreed minimum amount of money termed as “savings” during their regular meeting (Fig. 2). The savings are then pooled and members can take interest-bearing loans from this pool based on demand. This goes on for a period of 8–10 months when the lending stops and outstanding loans are repaid in readiness for share out. Share out, which is paying back of savings together with the interest gained from loaning, is done during the last month of the annual cycle. At the end of the cycle, the savings are paid back, and earned interest is distributed to all members proportional to the amounts saved. It is also during this meeting that decisions are made about group membership during the next cycle. Other models of surveyed groups do not share out the savings during their share-out. Instead, they use these funds to make group investments and then share out the income generated over the cycle. Other surveyed groups engage in the merry-go-round concept, where members of the group contribute a fixed amount for a fixed duration and each member is paid the entirety of the collected money on a rotating schedule.

Membership for the next cycle is largely informed by a member’s financial record including their ability to save and repay their loan on time. Loan repayment was mentioned as a key contributor to group conflict and disintegration. Different groups handle loan defaulters differently; some groups discontinue defaulters’ membership, while others take legal action against them, and yet others retain them in the group for the purposes of recovering the money. Understanding group cycles and how loan defaulters are handled is important for developing protocols for handling participant retention and loss to follow-up during the trial. Understanding group dynamics and changing group composition is also necessary for hypothesis testing during the trial, given that these dynamics may impact the financial success of a group as well as the willingness of group members living with HIV to continue their HIV care.

#### Meeting frequency and location

Less than half (40%) of the groups reported monthly meetings; with a considerable number reporting inconsistent meeting schedules during certain time periods. Overall, inconsistencies were reported during holiday periods and during periods of planting and harvesting. Some groups also met less frequently during low fishing seasons due to reduced income among members and the need to channel available funds towards school fees and farming rather than microfinance.

The groups reported meeting in different venues. The common meeting location is the group members’ homesteads with majority of such groups having rotational meetings from one member’s home to another and a few meeting routinely in one member’s home. Other groups reported meeting at health facilities and this had three dimensions. One, groups that meet at a local health facility that does not provide HIV care services did so as a measure of protecting individual members HIV status and ultimately avoiding stigma. This was especially true for predominately HIV-positive groups in Trans Nzoia County. Two, some groups meet at a local facility that provides HIV care services even though group members do not necessarily receive their HIV care services in that health facility. Three, groups that meet at a mid-volume or high-volume HIV care facility where the members receive their HIV care. For such groups, they work with the health facility to align their group meetings with their HIV care appointment dates. Mid- and high-volume HIV care facilities are those with a patient population of 500–999 patients and more than 1000 patients, respectively.

### Mapping group meeting location in relation to HIV care health facilities

#### Busia County

**Fig. 3:**
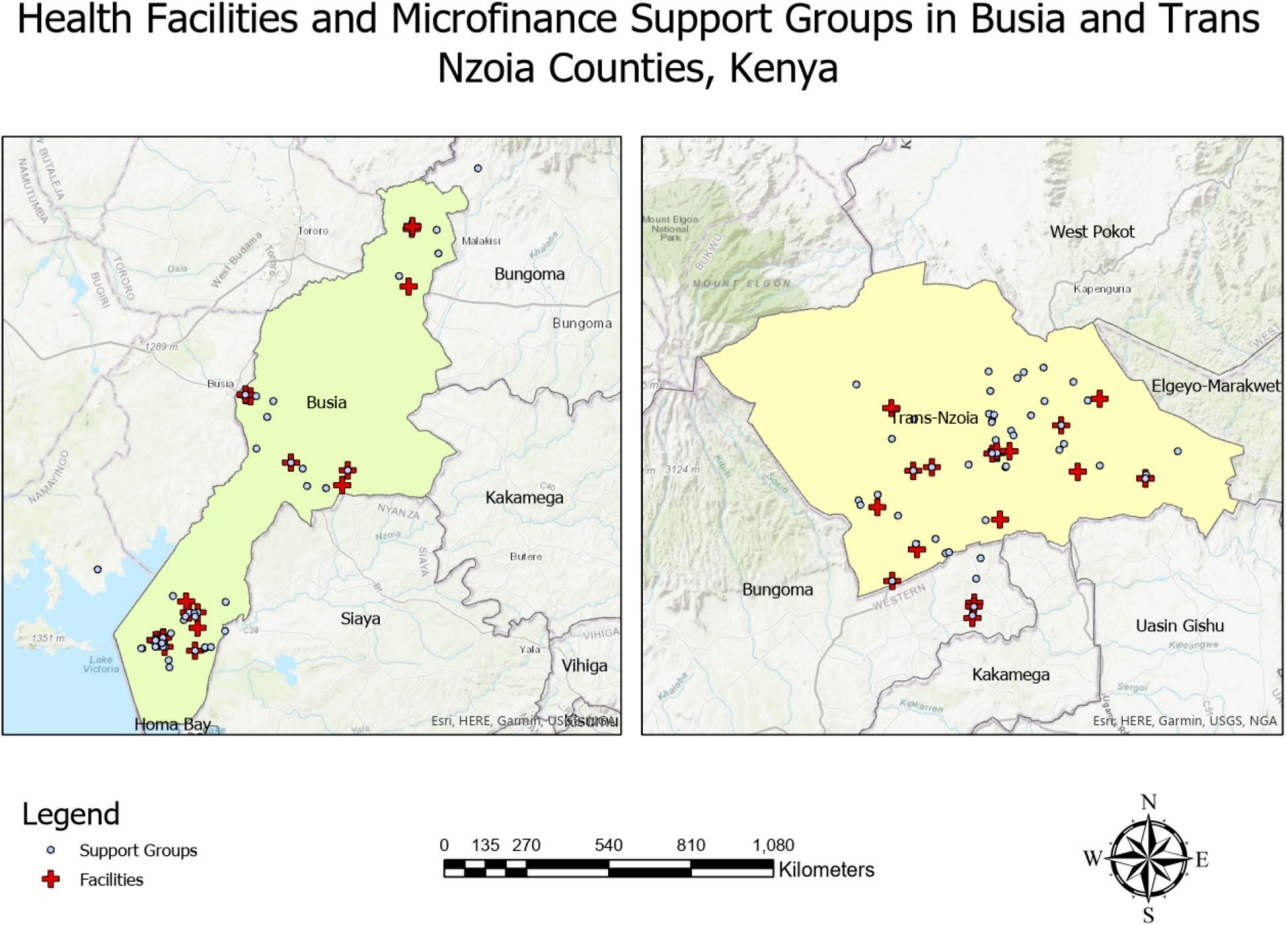
Locations of microfinance group meetings and health facilities where group members receive HIV care

Busia County has 45 AMPATH HIV clinics. Surveyed groups identified seven facilities where most of their members seek HIV care services. One facility, Port Victoria Sub-County Health Facility, was reported as the facility where about a third (32%) of the groups’ members seek HIV care. All seven facilities mentioned were either mid-volume or high-volume HIV facilities. The geospatial data revealed that groups in Busia County are concentrated around the HIV care facilities (Fig. 3) county, the mean distance from the group meeting location to the facility where a majority of members receive HIV care in Busia is 2.84 miles (SD 3.15 miles).

#### Trans Nzoia County

Among the 55 AMPATH HIV clinics in Trans Nzoia County, 12 were identified as the facilities were group members seek their HIV care with Kitale County Referral Hospital being reported as the health facility were half (50%) of the groups had their members seeking HIV care. This was largely out of fear of being spotted at HIV health facility located near them by people known to them. This has a cost and time implication on these patients as they are required to have regular contact with their HIV care providers. As illustrated in Fig. 3, groups meeting locations are widely spread out with majority of the groups meeting in locations situated far from the HIV care facilities. The mean distance from the groups meeting location to the health facility where group members seek HIV care is 3.25 miles and ranges from 15.52 to 0 miles (Table 4).

**Table 4:** Distance from group meeting location to HIV care facility

Distance (miles)	Mean	SD	Min	Max
Busia County	2.84	3.15	0	18.32
Trans Nzoia County	3.25	3.32	0	15.52

*SD* standard deviation

In both counties, we found that the majority of group members seek HIV care services at high-volume (53%) or mid-volume (32%) AMPATH health facilities. Mapping the groups’ meeting locations in relation to their health facilities is critically important to ensuring smooth logistical operations for the clinical teams who will be based at the relevant health facilities.

#### Smartphone ownership

Of the eligible groups surveyed, 64% had at least one member who owned a smartphone. Having access to smartphone technology would enable the groups to use apps and mobile banking services for tracking their group finances. In addition, given the challenges associated with COVID-19, having members connected by smartphone would enable continuation of many group activities during social distancing measures. Assessment of smartphone ownership is key to informing decisions on how group microfinance data will be collected and entered into data management platforms during the trial.

### Acceptability and suitability of the intervention and study procedures

We held 7 face-to-face stakeholder meetings and 2 stakeholder workshops to determine the acceptability and suitability of the intervention and study procedures for the local context, as well as to identify key implementation considerations (Table 1). Minutes from these meetings and workshops together with field notes from the survey were organized into two categories: (1) stakeholder perception of the intervention and (2) stakeholder recommendations for adapting the intervention.

#### Perception of intervention

Group leaders, GESPs, CHVs, and the key stakeholders in the health system expressed enthusiasm and support for the intervention. At the facility level, the intervention was perceived as having the potential to significantly improve patient’s retention in care and viral load suppression as it addresses barriers related to distance, congestion at the clinic and provider-patient relationship dynamics. Furthermore, this intervention provides for community viral load testing, an innovation that the AMPATH care team expressed desire to learn more about and possibly adopt in the future so as to fully achieve a community differentiated care model. The current AMPATH differentiated care model requires patients to visit the health facility annually or semi-annually for purposes of viral load testing.

At the community level, the intervention was perceived as additional support to HIV patients. Group leaders GESPs and CHVs reported that there are rising cases of non-communicable diseases in the community thus the idea of a community-based integrated care model was highly welcomed. Furthermore, the frequent group visits by health care providers is perceived as an opportunity to closely monitor HIV patients and to offer groups education on HIV management to dispel prevailing myths and misconceptions.

#### Recommendations for the intervention

Minutes from the meetings and workshops, as well the field notes further revealed some implementation considerations for the intervention. Key stakeholders expressed a desire to have this intervention integrated into the AMPATH care model for ease of transition after the study period. This, they said, can only be realized through continuous engagement of various stakeholders at the county and headquarters levels, collaboration on various aspects of the study, and overall increased transparency. Additionally, key stakeholders pointed out the need to maximize on the potential of the intervention, potential challenges to the intervention and recommendations for overcoming these challenges.

##### Opportunities to collaborate

Collaboration was viewed as an avenue for fostering local ownership of the integrated care model, which would ultimately influence the longevity of the intervention. Rather than developing study-specific protocols in a silo and to avoid creating a parallel program, the Harambee trial was urged to use existing structures such as established National HIV and NCD protocols, AMPATH-employed motorcycle riders for transporting blood samples, AMPATH-employed pharmaceutical technologists, and AMPATH’s laboratory for sample analysis. The study was further urged to employ a clinical team with experience working within the AMPATH care program. This team together with all other study employees working at the county level will report to the AMPATH county administrators and project manager.

##### Maximize on the intervention potential

Group leads gave suggestions on maximizing the potential of this intervention. On the health care component, they suggested inclusion of cancer screening and especially cervical cancer within this community-based integrated care model. While on the microfinance component, they reported not feeling adequately equipped to make best use of their group savings despite having received some form of training on microfinance management. They therefore suggested that the intervention provides training and mentorship on various aspects: predominantly group investment, investment diversification and handling defaulters. This information is crucial in the development of financial literacy training materials that are reflective and more responsive to the needs of the target groups. These financial literacy sessions which will be conducted throughout the 18-month intervention period, will be designed to fill in the notable gap in group knowledge, skills, and efficacy related to managing and controlling finances. This will not only enhance the groups’ capacity for saving and/or investing but also their retention in the study, especially for the control arm participants.

##### Potential challenges

Stakeholders flagged areas that could potentially pose challenges to the intervention. Firstly, facility in charges, program officers and county administrators expressed concerns over the microfinance component in community-based HIV care groups. This, they perceived, has had a negative influence on patient’s HIV care in the past with patients who default on repaying their loans in such groups dropping out of HIV care in fear of being tracked at the health facility by group members. Secondly, there were fears among county administrators and county Medical Officers that the study might encroach on existing studies and/or partner projects. However, meetings with these groups revealed that partner projects were targeting a different category of HIV patients while the chronic disease management (CDM) studies were focusing on health system strengthening through empowering local health facilities to provide CDM. Thirdly, stakeholders were not confident with clinicians’ ability to draw blood for VL testing as it had been proposed in the study protocol. This they attributed to clinician’s lack of experience in this area. They recommended having phlebotomists as part of the study’s clinical team. Finally, there were concerns about the study’s inclusion/exclusion criteria with stakeholders urging the study to follow National HIV Guidelines on Community-based ART Groups. These guidelines state that virally unsuppressed patients and pregnant women should be exempted from the community-based ART groups. They further urged the study team to widen its scope to include PMTCT mothers as well as children and adolescents living with HIV and their caregivers. They attributed this to the fact that caregivers and children living with HIV are normally given the same return-to-clinic date thus having a caregiver receive HIV care at the community level and then bring the child to the health facility undermines the efforts of community-based HIV care.

## Discussion

This feasibility study was conducted in preparation for a cluster randomized trial and brought to light a range of implementation issues that need to be addressed prior to testing the integrated HIV/NCD care model in the community (Table 5). The feasibility work confirmed the appropriateness of some of the original trial implementation strategies such as use of established HIV and NCD care protocols and reliance on trained clinical officers. However, this work also suggested several important changes to the study design.

**Table 5:** Implications of the feasibility assessment on the Harambee cluster randomized trial design

Concept	Original study criterion	Barrier identified during feasibility study	Opportunity identified during feasibility study	Criterion adaptation
Inclusion criteria (group-level)	Predominantly HIV-positive GISE groups	GISE groups have either evolved into mixed groups or turned dormant	____	Opening up the intervention to community-based HIV groups that engage in microfinance activities
Inclusion criteria (individual-level)	HIV-positive adults	National HIV guidelines outline patient’s eligibility for participation in community-based ART groups	____	(1) HIV-positive. However should: - Be virally suppressed - Not be pregnant - Not be a member of a CAG - Have no active opportunistic infection/other serious comorbidity such as cancer. (2) Development of a protocol on handling virally unsuppressed participants
Definition of active membership at the group level	Have participated in at least one microfinance group meeting in the prior 12 months at study baseline	Groups define active membership differently	_____	Have participated in at least one microfinance group meeting in the prior 6 months at study baseline
Clinical team	Team to comprise of clinical officers, pharmaceutical technologists, social workers and peer mentors, on a full-time basis.	Low confidence in clinicians’ ability to adequately conduct some procedures such as drawing blood for viral load testing.	(1) Study to rely on existing structures such as the county-based riders program and CHVs.	(1) Study to employ phlebotomists. (2) Study to work with the county-based riders on sample collection and delivery.

*GISE* Group Integrated Savings for Empowerment [interchangeably also called microfinance groups], *CAG* community ART group, *CHVs* community health volunteers

Our feasibility objective of determining our recruitment capability and resulting sample characteristics revealed the need for the study to review its inclusion criteria at both the group and individual levels. At a group level, the original intent of the study was to enroll predominately HIV-positive GISE groups. However, the feasibility study revealed that most of these groups have either evolved into mixed groups or turned dormant with very few GISE groups meeting the study inclusion criteria. The study therefore expanded to include community-based HIV groups that engage in any type of microfinance activities. Furthermore, we found variations in the definitions of active membership at an individual level in the group. This information led to a single, standardized definition by the study team.

Meetings with the various stakeholders revealed the need to adapt the most recent national HIV and NCD care delivery protocols. This had an implication on the study procedures around handling unsuppressed patients. The study had proposed to provide interventions for virally unsuppressed in the community-based care. However, key stakeholders made it apparent that this goes against the latest clinical guidelines for handling unsuppressed patients. This led to the development of a protocol on referring participants who are virally unsuppressed at baseline and those who become unsuppressed in the course of the study to the clinic for follow-up testing and additional adherence counseling, as they would have in clinic-based care.

The study had initially proposed to hire a clinical cadre comprised of clinical officers, pharmaceutical technologists, social workers and peer mentors to serve the study on a full-time basis. The feasibility work revealed, however, the need for a larger team comprised of AMPATH-affiliated personnel, and the study will now employ clinical officers, phlebotomists and FPI officers as suggested. As part of collaboration with the AMPATH care program, the study will use the program’s pharmaceutical technologists and county-based riders for transportation of blood samples. Additionally, the majority of groups were lacking adequate financial management skills with groups highlighting areas in which they need support. This information is important for the development of financial literacy materials that will be used to empower the groups through continuous training and mentorship by the FPI officers on the team. Also, prior to this feasibility assessment, we had planned for microfinance group leaders to implement an app-based bookkeeping system using their smart phones to help manage their group’s saving and lending in a streamlined way, and for researchers to access microfinance data remotely via this application. However, this assessment indicated that reliance on leader’s smart phones for data collection would be difficult because leadership positions are held on rotational basis and training leaders in the application would be resource intensive.

Some of the issues brought to light by the feasibility study were beyond the scope of the proposed intervention, and thus will not be considered. These were issues raised by stakeholders in their quest to have the study provide a holistic integrated model. These include the suggested inclusion of procedures such as cancer screening as well as the inclusion of more categories of HIV patients such as PMTCT mothers, and children and adolescents living with HIV. Additionally, we acknowledge that the microfinance groups included in this feasibility study may not be representative of microfinance groups in other catchment areas of Western Kenya or other Sub-Saharan African countries in terms of the income level and self-agency of group members. The Harambee trial will enroll existing, self-sustaining microfinance groups to test the integrated care intervention and thus has limited ability in selecting microfinance group members based on specific socioeconomic criteria (e.g., the poorest of the poor) [22].

## Limitations

This study was not without limitations. We used workshops and in-person meetings to generate recommendations from multi-sectoral stakeholders [46] but acknowledge that it is difficult to establish consensus via this approach. Despite this, we believe our ability to survey more than 100 rural microfinance group leaders and to synthesize survey data with geospatial and qualitative findings provided highly valuable insights into the feasibility of implementation our clinical trial.

## Conclusion

This feasibility study conducted in Western Kenya confirmed that there is an urgent need for studies that provide robust evidence of novel, differentiated care models that address the dual burden of HIV and NCDs in the community. Our feasibility findings highlight the need for refinement of the study inclusion criteria to align with community-level norms, allowing some flexibility in care protocols across to provide for variability across local health facilities, and having researchers take a participatory approach to facilitate collaboration and engagement with key stakeholders at each stage of the trial. For future community-based interventions, we recommend that researchers assess feasibility both prior to trial implementation and on an ongoing basis to determine the appropriateness of a given intervention and to amend the intervention in final planning and early implementation stages.

## Supplementary Information

**Additional file 1: Appendix 1.** Harambee Project Mapping Tool 2019.

## Data Availability

All data generated or analyzed during this study are included in this published article. The datasets used and/or analyzed during the current study are available from the corresponding author on reasonable request.

## Abbreviations

AMPATH: Academic Model Providing Access To Healthcare
ART: Antiretroviral therapy
CAG: Community ART group
CDM: Chronic disease management
CHV: Community health volunteer
FPI: Family preservation initiative
GESP: Group empowerment service provider
GISE: Group integrated savings for empowerment
ICB: Integrated community-based care
MF: Microfinance
NCD: Non-communicable disease
PLHIV: People living with HIV
PMTCT: Prevention of mother-to-child transmission
