# Supplementary material for: Integrating community-based HIV and non-communicable disease care with microfinance groups: a feasibility study in Western Kenya
Source: Pilot Feasibility Stud. 2022 Dec 28;8:266. doi: 10.1186/s40814-022-01218-6 (PMC9795156; doi:10.1186/s40814-022-01218-6)
Supplement: Supplementary file 1 — Additional file 1: Appendix 1. Harambee Project Mapping Tool 2019. [file 40814_2022_1218_MOESM1_ESM.docx]

1. County
   - Busia
   - Trans Nzoia
   - Other (Specify)______________
2. Name of the Group

- ______________

1. When was this group formed? _________________________________

- Day/Month/Year

1. Total number of members: ________________________________________________________

- Active members: __________________________________________________________
- Inactive members: ________________________________________________________
- Other (Specify): ___________________________________________________________

1. Who is an active member? ____________________________________________________________________________________________________________________________________________________________________________________________________________________________________________________________________________________________________________________________________________________________________________________________________________________________________________________________________________________
2. How often do you meet as a group?

- Weekly
- Bi-Monthly
- Monthly
- Quarterly
- Other (Specify)______________________________________

1. Where do you mostly meet? ______________________________________________________

- GPS Co-ordinates of meeting Location ________________________________________

1. When was the last time you met as a group? ____________________________________

- Date/Month/Year
- Other (Specify)_________________________________________

1. When was the last time you had a financial activity going on within the group (Saving, Loaning, etc.)

- Date/Month/Year_______________________________
- Other (Specify)____________________________________

1. How many members of this group are receiving HIV care? _______________________________
2. How many members of this group have disclosed their HIV status to the group?

- Number______________
- Don’t know

1. Where do most members of this group go for HIV care? _________________________________

- GPS co-ordinates of the health facility_________________________________________

1. Has your group ever participated in other research studies such as BIGPIC study?

- Yes
- No
- Not sure

1. Who is the Chairman of this group?

- Name: _____________________
- Phone No: _________________

1. Who is the Secretary of this group?

- Name: _____________________
- Phone No: _________________

1. Who is the Treasurer of this group?

- Name: _____________________
- Phone No: _________________

1. How many members in your group own a smart phone? ________________________________

Questions: ____________________________________________________________________________________________________________________________________________________________________________________________________________________________________________________________________________________________________________________________________________________

Additional comments: ____________________________________________________________________________________________________________________________________________________________________________________________________________________________________________________________________________________________________________________________________________________

Recommendations: ____________________________________________________________________________________________________________________________________________________________________________________________________________________________________________________________________________________________________________________________________________________
